# Suppression of Alzheimer’s Disease-Related Phenotypes by Geranylgeranylacetone in Mice

**DOI:** 10.1371/journal.pone.0076306

**Published:** 2013-10-01

**Authors:** Tatsuya Hoshino, Koichiro Suzuki, Takahide Matsushima, Naoki Yamakawa, Toshiharu Suzuki, Tohru Mizushima

**Affiliations:** 1 Faculty of Pharmacy, Keio University, Minato-ku, Tokyo, Japan; 2 Graduate School of Pharmaceutical Sciences, Hokkaido University, Sapporo, Hokkaido, Japan; "Mario Negri" Institute for Pharmacological Research, Italy

## Abstract

Amyloid-β peptide (Aβ) plays an important role in the pathogenesis of Alzheimer’s disease (AD). Aβ is generated by the secretase-mediated proteolysis of β-amyloid precursor protein (APP), and cleared by enzyme-mediated degradation and phagocytosis. Transforming growth factor (TGF)-β1 stimulates this phagocytosis. We recently reported that the APP23 mouse model for AD showed fewer AD-related phenotypes when these animals were crossed with transgenic mice expressing heat shock protein (HSP) 70. We here examined the effect of geranylgeranylacetone, an inducer of HSP70 expression, on the AD-related phenotypes. Repeated oral administration of geranylgeranylacetone to APP23 mice for 9 months not only improved cognitive function but also decreased levels of Aβ, Aβ plaque deposition and synaptic loss. The treatment also up-regulated the expression of an Aβ-degrading enzyme and TGF-β1 but did not affect the maturation of APP and secretase activities. These outcomes were similar to those observed in APP23 mice genetically modified to overexpress HSP70. Although the repeated oral administration of geranylgeranylacetone did not increase the level of HSP70 in the brain, a single oral administration of geranylgeranylacetone significantly increased the level of HSP70 when Aβ was concomitantly injected directly into the hippocampus. Since geranylgeranylacetone has already been approved for use as an anti-ulcer drug and its safety in humans has been confirmed, we propose that this drug be considered as a candidate drug for the prevention of AD.

## Introduction

Alzheimer’s disease (AD) is the most common neurodegenerative disorder and the leading cause of adult-onset dementia. AD is characterized pathologically by the accumulation of neurofibrillary tangles and senile plaques, the latter of which are composed of amyloid-β peptide (Aβ), such as Aβ40 and Aβ42 [1]. To generate Aβ, β-amyloid precursor protein (APP) is first cleaved by β-secretase and then by γ-secretase [2]. γ-secretase is composed of four core components, including presenilin (PS)1 and PS2 [3]. Since early-onset familial AD is linked to three genes, *app, ps1* and *ps2* [3], Aβ is believed to be a key factor in the pathogenesis of AD. Monomeric Aβ easily self-assembles to form oligomers, protofibrils and fibrils, with the oligomers and protofibrils being more neurotoxic than other forms of Aβ [4]. Aβ can be cleared from the brain by different mechanisms such as degradation by enzymes (neprilysin, insulin-degrading enzyme (IDE) and endothelin-converting enzyme (ECE)-2) and phagocytosis by microglia and astrocytes [5]. Therefore, cellular factors that affect the production, self-assembly and clearance of Aβ are likely to offer appropriate targets for the development of drugs to prevent or treat AD.

Inflammation is also important in the pathogenesis of AD [6]. We have previously reported that prostaglandin E_2_ (PGE_2_), a potent inducer of inflammation, enhances the production of Aβ [7-9], suggesting that inflammation is an aggravating factor for AD. However, as inflammation also activates the Aβ phagocytotic activity of microglia and astrocytes [10], it is evident that the relationship between inflammation and AD progression is complex. Further to the above, transforming growth factor (TGF)-β1, a key cytokine regulating the brain’s response to injury and inflammation, was reported to suppress the progression of AD by stimulating microglial Aβ clearance [10,11].

Cellular up-regulation of expression of heat shock proteins (HSPs), particularly that of HSP70, provides resistance to stressors through the process of refolding or degrading denatured proteins produced by the stressors [12]. Furthermore, several studies have reported that intracellular HSP70 displays anti-inflammatory activity [13,14]. Therefore, HSP70 has received considerable attention for its therapeutic potential. For example, we have shown, using transgenic mice overexpressing HSP70, that HSP70 protects against the development of gastric and small intestine-related lesions, inflammatory bowel disease-related colitis, pulmonary fibrosis and ultraviolet-induced skin damage and hyperpigmentation [15-20].

A number of previous studies have suggested that the expression of HSPs, in particular HSP70, could suppress the progression of AD (see below). Some part of HSPs is secreted into the extracellular space and HSP70 and HSP90 recognize Aβ oligomers and decrease the level of Aβ self-assembly, resulting in the suppression of the production of toxic Aβ [21-23]. Artificial expression of HSP70 protects cultured neurons from Aβ-induced apoptosis [24]. HSP70 stimulates the degradation of APP and Aβ *in vitro* [25], and it has been shown that purified HSP70 and HSP90 activate the phagocytotic activity for Aβ [26]. Furthermore, we showed that crossing APP23 mice (used as an animal model for AD) with transgenic mice overexpressing HSP70 suppresses both the functional and pathological phenotypes. This outcome is probably due to HSP70’s activities of anti-aggregation, neuroprotection and stimulation of Aβ clearance [27], and suggests that inducers of HSP70 expression could be good candidates as drugs to treat or inhibit the progression of AD.

Geranylgeranylacetone (GGA), a leading anti-ulcer drug on the Japanese market, has been reported to be a non-toxic HSP-inducer [28]. The HSP-inducing activity of GGA is a major component of its gastro-protective activity [15,17]. Through its cytoprotective, anti-inflammatory and anti-aggregation activities, the induction of HSP70 expression by GGA shows ameliorative effects in animal models of various diseases, such as small intestine-related lesions, inflammatory bowel diseases and pulmonary fibrosis [16,29]. In this study, we examined the effect of GGA on AD phenotypes exhibited by APP23 mice. Orally administered GGA improved not only cognitive deficits displayed by these animals, but also the pathological manifestations associated with these phenotypes.

## Materials and Methods

### Materials

The fluorescent substrate for β-secretase (H2N-Arg-Glu-(EDANS)-Glu-Val-Asn-Leu-Asp-Ala-Glu-Phe-Lys-(DABCYL)-Arg-OH) was purchased from Calbiochem (Dermstadt, Germany), while fluorescent substrate for γ-secretase (Nma-Gly-Gly-Val-Val-Ile-Ala-Thr-Val-Lys(Dnp)-D-Arg-D-Arg-D-Arg-NH2) and synthetic Aβ42 were from Peptide Institute (Osaka, Japan). Alexa Fluor 488 goat anti-mouse immunoglobulin G was purchased from Invitrogen (Carlsbad, California). Sandwich ELISA kit to detect Aβ oligomers or those to detect Aβ40 and Aβ42 were from Immuno-Biological Laboratories (Fujioka, Japan) or Wako (Osaka, Japan), respectively. The antibody to actin was from Santa Cruz (Santa Cruz, California). Thioflavin-S and antibodies to the C-terminal fragment (CTF) of APP and synaptophysin were from Sigma (St. Louis, Missouri). The antibody to HSP70 was from R&D systems (Minneapolis, MN) and that to PS1 was from Chemicon (Temecula, California). The RNeasy kit was obtained from Qiagen (Valencia, California). The PrimeScript® 1st strand cDNA Synthesis Kit was from TAKARA Bio (Ohtsu, Japan) and SsoFast^TM^ EvaGreen Supermix was from Bio-Rad Laboratories (Hercules, California). Mounting medium for immunohistochemical analysis (VECTASHIELD) was from Vector Laboratories (Burlingame, California). Mounting medium for histological examination (malinol) was purchased from Muto Pure Chemicals (Tokyo, Japan). The Envision kit was from Dako (Carpinteria, California). GGA was purchased from Eisai (Tokyo, Japan).

### Animals and Drug administration

APP23 mice (C57BL/6 mice expressing mutant APP (Swedish type)) were a gift from Dr. M Staufenbiel (Novartis Institutes for BioMedical Research, Basel, Switzerland) [30]. All experiments in this study were performed using littermate female mice (heterogeneous APP23 and wild-type mice).

For repeated oral administration of GGA to mice, we used GGA-supplemented chow. GGA granules were mixed with powdered rodent chow at a concentration of 1%. On the assumption that the average mouse weighs 24 g and consumes 3 g of chow per day, this dose was predicted to supply 1.25 g GGA per kg body weight per day. The actual average dose of GGA was calculated to be 1.76 g per kg body weight per day based on the amount of chow taken. The LD_50_ value of GGA (a dose causing 50% survival rate) in mice was reported to be more than 15 g per kg body weight [31].

For the single oral administration of GGA to mice, GGA was dissolved in 5% gum Arabic and 0.06% Tween and administered orally (500 mg/kg, 10 ml/kg).

The experiments and procedures described here were carried out in accordance with the Guide for the Care and Use of Laboratory Animals as adopted and promulgated by the National Institutes of Health, and were approved by the Animal Care Committee of Keio University (Permit Number: 12001-0). All surgery was performed under sodium pentobarbital anesthesia, and all efforts were made to minimize suffering.

### Preparation and Injection of Aβ42 oligomers

Oligomerized Aβ42 was prepared as described previously [32], with minor modifications. Aβ42 peptides were dissolved in 1, 1, 1, 3, 3, 3-hexafluoro-2-propanol, dried and stored. The dried peptides were dissolved in anhydrous DMSO at 100 mM and diluted in saline to give a final concentration of 100 µM. After 16 h incubation at 22°C, the preparation was centrifuged at 21, 000 x g for 15 min and the supernatant was used for experiments.

For the injection of Aβ42 oligomers, mice were anaesthetized with pentobarbital sodium. Bilateral stereotaxic injection of Aβ42 oligomers (1 µl, 100 pmol) or saline (sham) into the hippocampus (AP -2.5mm, L +/-2.0 mm, DV -1.5 mm) was performed using a Hamilton syringe. The injection speed was 0.5 µl/min and the needle was maintained in place for an additional 2 min before being slowly withdrawn. The dose and procedures for the injection were determined based on previous papers [33,34].

### Immunoblotting Analysis

Whole cell extracts were prepared as described previously [27]. The protein concentration of each sample was determined by the Bradford method. Samples were applied to SDS polyacrylamide gels (Tris/tricine gels for the detection of CTFα and CTFβ, and Tris/glycine gels for the detection of other proteins) and subjected to electrophoresis, after which proteins were immunoblotted with each antibody.

### Real-time RT-PCR Analysis

Real-time RT-PCR was performed as described previously [27], with some modifications. Total RNA was extracted from the brain using an RNeasy kit according to the manufacturer’s protocol. Samples (1 µg RNA) were reverse-transcribed using a first-strand cDNA synthesis kit. Synthesized cDNA was used in real-time RT-PCR (Chromo 4 instrument, Bio-Rad Laboratories) experiments using SsoFast^TM^ EvaGreen Supermix, and then analyzed with Opticon Monitor Software. Specificity was confirmed by electrophoretic analysis of the reaction products and by inclusion of template- or reverse transcriptase-free controls. To normalize the amount of total RNA present in each reaction, glyceraldehyde-3-phosphate dehydrogenase (GAPDH) cDNA was used as an internal standard.

Primers were designed using the Primer3 website. The primers used were (name: forward primer, reverse primer): *gapdh*: 5’-aactttggcattgtggaagg-3’, 5’-acacattgggggtaggaaca-3’; *neprilysin*: 5’-gcagcctcagccgaaactac-3’, 5’-caccgtctccatgttgcagt-3’; *ide*: 5’-accaggaaatgttggctgtc-3’, 5’-tctgagaggggaactctcca-3’; *ece-2*: 5’-gctatgcccatgtacccagt-3’, 5’-tggcatccagagtacccttc-3’; *il-1β*: 5’-gatcccaagcaatacccaaa-3’, 5’-ggggaactctgcagactcaa-3’; *il-6*: 5’-ctggagtcacagaaggagtgg-3’, 5’-ggtttgccgagtagatctcaa-3’; *tnf-*α: 5’-cgtcagccgatttgctatct-3’, 5’-cggactccgcaaagtctaag-3’; *tgf-β1*: 5’-tgacgtcactggagtacgg-3’, 5’-ggttcatgtcatggatggtgc-3’.

### Morris Water Maze Test

The Morris water maze test was conducted in a circular 90 cm diameter pool filled with water at 22.0 + 1°C, as described previously [9]. In the hidden platform test, a circular platform (10 cm in diameter) was submerged 0.5 cm below water level. Swimming paths were tracked for 60 s with a camera, and stored in a computer (Video Tracking System CompACT VAS/DV, Muromachi Kikai, Tokyo, Japan). The mice were given 4 trials (1 block) per day for 7 consecutive days, during which the platform was left in the same position. The time taken to reach the platform (escape latency) was measured and the average time for the 4 trials was determined.

Twenty-four hours after the last trial of the hidden platform test, the mice were subjected to a transfer test in which the platform was removed, and their swimming path was recorded for 60 s. Percent search time for each quadrant and crossing time in the area where the platform had been located were determined.

### Sandwich ELISA (sELISA) for Aβ

Aβ40 and Aβ42 levels in the brain were determined as described previously [35]. Briefly, the brain hemispheres were homogenized in 50 mM Tris/HCl buffer (pH 7.6) containing 150 mM NaCl, and then centrifuged. Guanidine/HCl (0.5 M, final concentration) was added to the supernatants (soluble fractions). The precipitates were solubilized by sonication in 6 M guanidine/HCl, after which the solubilized pellet was centrifuged to obtain supernatants (insoluble fractions). The amounts of Aβ40 and Aβ42 in each fraction were determined by sELISA according to the manufacturer’s instructions (Wako). An ELISA assay for Aβ oligomers was carried out on the soluble fractions (but without guanidine/HCl) according to the manufacturer’s instructions (Immuno-Biological Laboratories).

### Thioflavin-S Staining and Immunohistochemical Analyses

Brain hemispheres were fixed in 4% buffered paraformaldehyde and embedded in paraffin before being cut into 4 µm-thick sections, which were then deparaffinized and washed in phosphate-buffered saline. Sections were prepared from the coronal plane and images were carefully matched for location in the rostral-caudal (2.0 mm posterior to bregma) directions. Observers were blinded to conditions in all experiments. For statistical analysis, we used three sections (every 10 sections) per mouse and calculated an average value for the three sections. The *n* value reported in each figure indicates the number of mice used for experiments.

For thioflavin-S staining, sections were stained with a 1% thioflavin-S solution. Samples were mounted with malinol and inspected using a BX51 microscope (Olympus, Tokyo, Japan). Fluorescence microscope images of regions (1.0 mm^2^) in the hippocampus or cerebral cortex were used to calculate the area stained with thioflavin-S; this was done using LuminaVision software (Mitani, Fukui, Japan). Using the threshold optical density, we divided the total area under consideration into thioflavin-S-positive and negative areas and determined the thioflavin-S-positive area as a percentage of the total area.

For immunohistochemical analysis to detect synaptophysin, sections were blocked with 2.5% goat serum for 10 min, incubated for 12 h with antibody to synaptophysin (1:200 dilution) in the presence of 2.5% bovine serum albumin, and then incubated with Alexa Fluor 488 goat anti-mouse immunoglobulin G. Samples were mounted with VECTASHIELD and inspected with the aid of a BX51 fluorescence microscope. Fluorescence intensity in a 100 µm x 150 µm area of the hippocampal CA3 region was determined using LuminaVision software and expressed relative to the fluorescence intensity measured in the same region in wild-type mice.

### β- and γ-secretase-mediated Peptide Cleavage Assay

β- and γ-secretase activity was monitored as reported previously [9]. Solubilized membranes were incubated for 1 h at 37°C in 200 µl of 50 mM acetate buffer (pH 4.1) containing 100 mM sodium chloride, 0.025% bovine serum albumin and 10 µM β-secretase fluorescent substrate, or for 4 h at 37°C in 200 µl of 50 mM Tris/HCl buffer (pH 6.8) containing 2 mM EDTA, 0.25% CHAPSO and 10 µM γ-secretase fluorescent substrate. Fluorescence was measured using a plate reader (Fluostar Galaxy, BMG Labtechnologies, Offenburg, Germany) with an excitation wavelength of 355 nm and an emission wavelength of 510 nm (for β-secretase) or 440 nm (for γ-secretase).

### Statistical Analysis

All values are expressed as the mean ± SEM. One- or two-way analysis of variance (ANOVA) followed by the Tukey test was used to evaluate differences between more than three groups. The Student’s *t*-test for unpaired results was used for the evaluation of differences between two groups. Differences were considered to be significant for values of *P*<0.05. The *P* value or *F* value of the ANOVA analysis are shown in the text.

## Results

### Effect of orally administered GGA on cognitive function in APP23 mice

**Figure 1:**
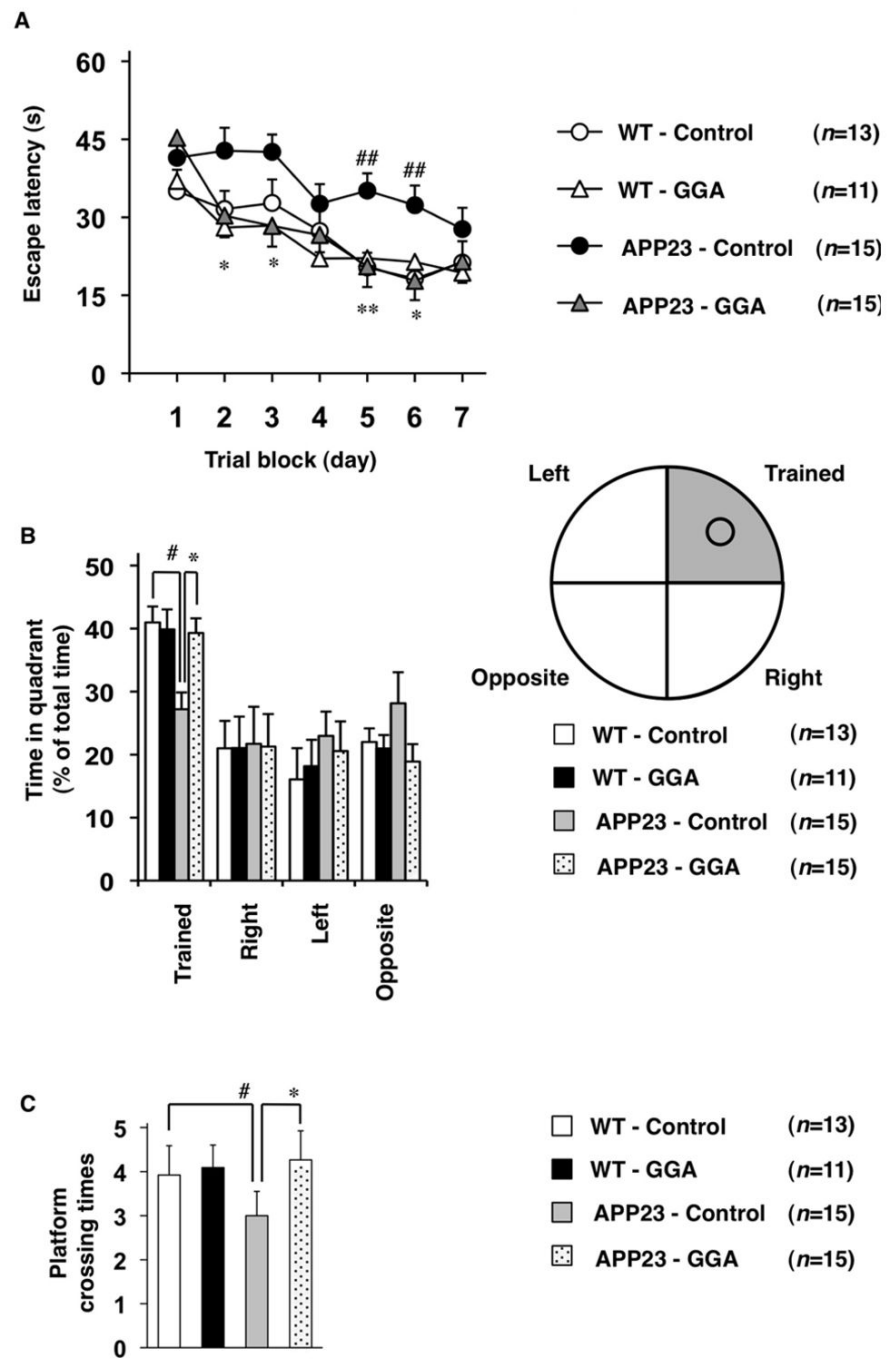
Effects of oral administration of GGA on spatial learning and memory in APP23 mice. Cognitive behavioral tests using the Morris water maze were carried out on 12-month-old wild-type (WT) and APP23 mice fed either GGA-supplemented chow (10 g GGA/kg chow) or control chow from 3 to 12 months of age as described in the experimental procedures. The average escape latency in each trial block (4 tests) was measured for 7 days (A), after which the mice were subjected to a transfer test in which the platform was removed. The spatial memory test for platform location was estimated by the percent search time spent in each quadrant (the platform had been located in the “trained” quadrant) (B) or platform crossing times (C). Values are given as mean ± SEM (*n*=13 for WT-Control, *n*=11 for WT-GGA, *n*=15 for APP23-Control, and *n*=15 for APP23-GGA). **P <0.01 and *P <0.05, APP23-GGA versus APP23-Control; ##P <0.01 and # P <0.05, APP23-Control versus WT-Control.

To examine the effect of GGA on APP23 mice at 12 months of age when AD-related phenotypes become apparent, we selected the administration period (9 months) and dosage (chow containing GGA at a concentration of 1%) based on a previous report [36]. The Morris water maze test was used to examine spatial learning and memory. APP23 and wild-type (C57BL/6) mice were fed either GGA-supplemented chow or control chow from the age of 3 months to 12 months and spatial learning and memory was tested at the end of this period (*n*=13 for WT-Control, *n*=11 for WT-GGA, *n*=15 for APP23-Control, and *n*=15 for APP23-GGA). No significant differences among the four groups of mice (APP23 or wild-type mice fed GGA-supplemented or control chow) were observed in terms of the amounts of chow consumed, in swimming speed or in ability to locate a visible platform (data not shown). Mice were trained 4 times per day for 7 days to learn the location of a hidden platform, and the time required to reach the platform (escape latency) was measured. As shown Figure 1A, APP23 mice fed control chow took a significantly longer time than wild-type mice on the same diet to reach the platform, confirming that APP23 mice have a deficiency in spatial learning and memory. APP23 mice fed GGA-supplemented chow took a significantly shorter time to find the hidden platform than their counterparts fed the control chow (Figure 1A). In contrast, GGA administration did not affect the escape latency in wild-type mice (Figure 1A) (two-way repeated measures ANOVA, genotype effect *F*_(1, 50)_=5.42, *P*=0.021; GGA effect *F*_(1, 50)_=4.92, *P*=0.031; interaction *F*_(1, 50)_= 4.09, *P*=0.048).

We next performed a transfer test to estimate the spatial memory of platform location. Following a 7-day training period (see above), each mouse was subjected to a Morris water maze test in which the platform was removed and the percent search time for each quadrant was measured. As shown in Figure 1B, the ratio of time spent in the trained quadrant was lower for the APP23 mice fed control chow compared not only with wild-type mice fed control chow but also with APP23 mice fed GGA-supplemented chow (Figure 1B) (Two-way ANOVA, genotype effect *F*_(1, 50)_=6.11, *P*=0.017; GGA effect *F*_(1, 50)_=3.87, *P*=0.055; interaction *F*_(1, 50)_=5.36, *P*=0.025). The crossing time of the area where the platform had been located, another indicator of spatial memory, was lower for the APP23 mice fed control chow compared with wild-type mice fed control chow and APP23 mice fed GGA-supplemented chow (Figure 1C) (two-way ANOVA, genotype effect *F*_(1, 50)_=3.64, *P*=0.062; GGA effect *F*_(1, 50)_=7.97, *P*=0.007; interaction *F*_(1, 50)_=5.76, *P*=0.020). GGA administration did not affect these indexes in wild-type mice (Figure 1B and C). These results suggest that the deficit in spatial learning and memory in APP23 mice was ameliorated by the oral administration of GGA.

### Effect of orally administered GGA on Aβ levels, Aβ accumulation and synaptic loss in APP23 mice

**Figure 2:**
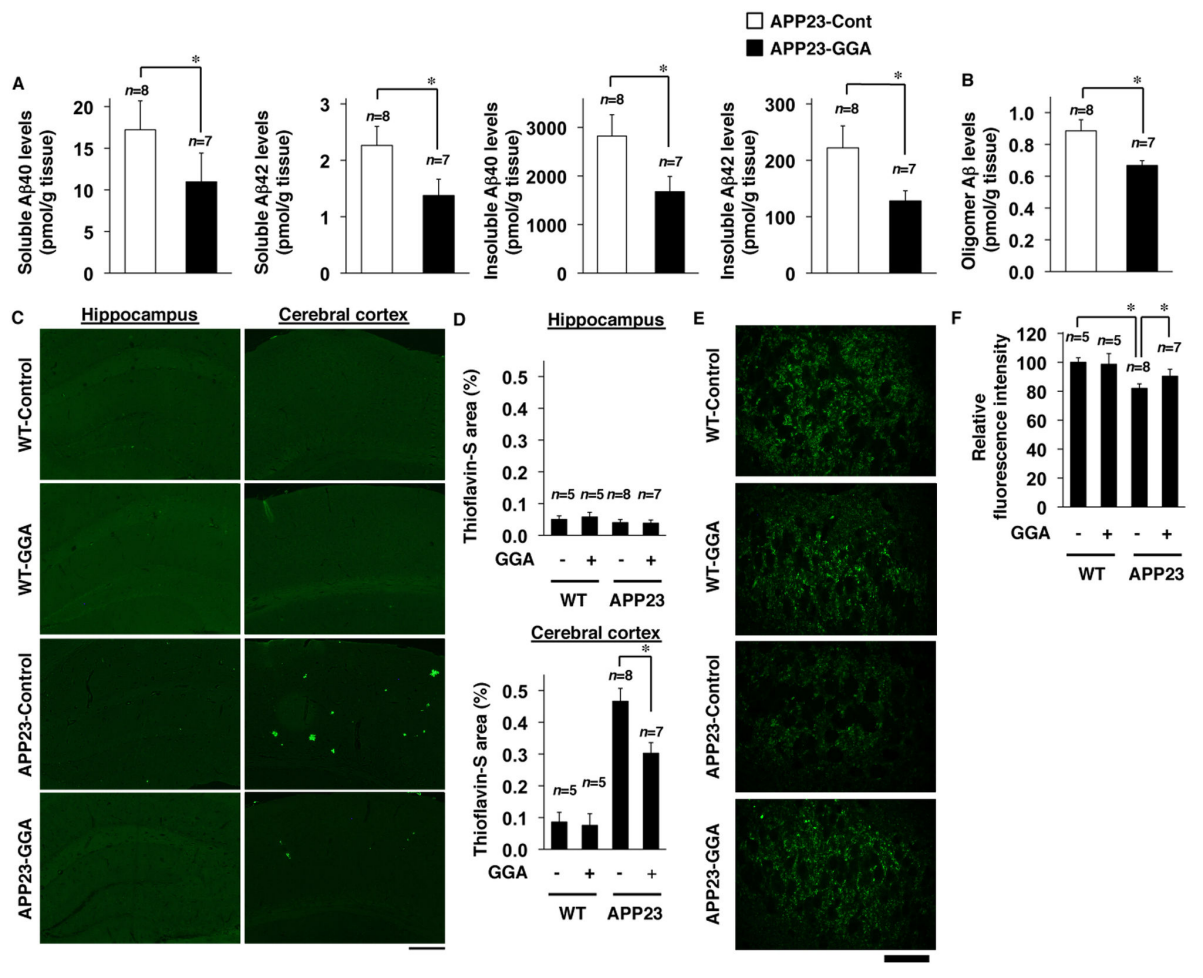
Effects of orally administered GGA on Aβ levels and synaptic loss. Soluble and insoluble fractions and sections were prepared from the whole brains of 12-month-old wild-type mice (WT) and APP23 mice fed either GGA-supplemented chow or control chow (see the legend of Figure 1). The amounts of Aβ40 and Aβ42 in each fraction or Aβ oligomers in the soluble fraction were determined by sELISA as described in the experimental procedures (A, B). Brain sections were subjected to thioflavin-S staining (scale bar, 200 µm) (C) or immunohistochemical analysis with an antibody to synaptophysin (scale bar, 50 µm) (E). The percentage of area stained with thioflavin-S in the hippocampus or cerebral cortex (D), or the relative fluorescence intensity (synaptophysin) in the hippocampal CA3 region (F) was determined. Three sections were prepared from each mouse and their average was calculated (D, F). Values are given as mean ± SEM (*n*=5 for WT-Control, *n*=5 for WT-GGA, *n*=8 for APP23-Control, and *n*=7 for APP23-GGA). **P<0.01; *P<0.05.

We have previously reported that genetic overexpression of HSP70 decreases levels of Aβ, Aβ plaque deposition and synaptic loss in mice [27]. In the present study, we examined whether similar alteration could be observed in APP23 mice administered GGA. Levels of Aβ40 and Aβ42 in both soluble and insoluble brain fractions were lower in APP23 mice fed GGA-supplemented chow than for those fed control chow (Figure 2A) (Student’s *t*-test, soluble Aβ40, *P*=0.046; soluble Aβ42, *P*=0.040; insoluble Aβ40, *P*=0.036; insoluble Aβ42, *P*=0.038). Similar results were observed with regard to the level of Aβ oligomers (Figure 2B) (*n*=8 for APP23-Control and *n*=7 for APP23-GGA) (Student’s *t*-test, *P* =0.011).

We next examined the effects of orally administered GGA on Aβ plaque deposition and neurotoxicity, which were reported to occur in APP23 mice [37]. Thioflavin-S staining revealed that the level of Aβ plaque deposition in the cerebral cortex was lower in APP23 mice fed GGA-supplemented chow than in those fed control chow (Figure 2C and D) (*n*=5 for WT-Control, *n*=5 for WT-GGA, *n*=8 for APP23-Control, and *n*=7 for APP23-GGA) (Figure 2D, upper panel, two-way ANOVA, genotype effect *F*_(1, 21)_=40.34, *P*<0.001; GGA effect *F*_(1, 21)_=10.49, *P*=0.004; interaction *F*_(1, 21)_=5.42, *P*=0.030). We could not detect to any significant extent the deposition of Aβ plaque in the hippocampus of APP23 mice at 12 months of age (Figure 2C and D) (Figure 2D, lower panel, two-way ANOVA, genotype effect *F*_(1, 21)_=0.25, *P*=0.620; GGA effect *F*_(1, 21)_=0.42, *P*=0.524; interaction *F*_(1, 21)_=0.14, *P*=0.710).

We also estimated the number of synapses based on synaptophysin staining and found that the level of staining was higher in sections from APP23 mice fed the GGA-supplemented chow compared with those fed control chow (Figure 2E and F) (*n*=5 for WT-Control, *n*=5 for WT-GGA, *n*=8 for APP23-Control, and *n*=7 for APP23-GGA) (two-way ANOVA, genotype effect *F*_(1, 21)_=7.84, *P*=0.011; GGA effect *F*_(1, 21)_=6.03, *P*=0.023; interaction *F*_(1, 21)_=6.81, *P*=0.016). These results suggest that synaptic loss was ameliorated by the administration of GGA. All results in Figure 2 suggest that the oral administration of GGA decreases the level of Aβ (monomer and oligomers) and Aβ plaque deposition in the brain and protects neurons against Aβ-induced neurotoxicity.

### Effect of orally administered GGA on the production of Aβ and on the expression of genes involving Aβ clearance

**Figure 3:**
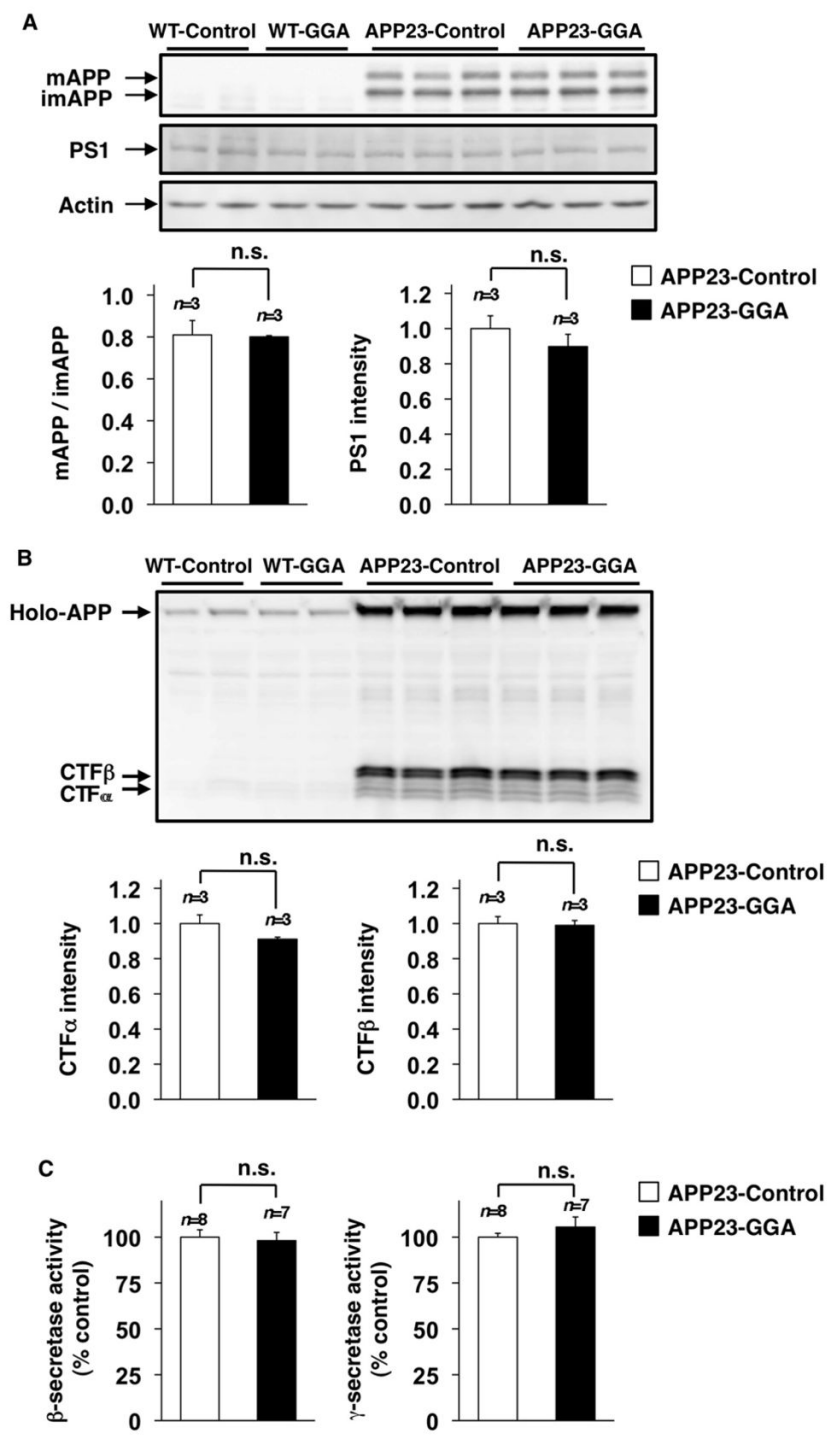
Effects of orally administered GGA on APP maturation and secretase activity. Whole cell extracts or membrane fractions were prepared from the brains of 12-month-old wild-type (WT) and APP23 mice fed either GGA-supplemented chow or control chow (see the legend of Figure 1). Whole cell extracts were subjected to immunoblotting with an antibody to APP (A, B), PS1 (A) or actin (A). The band intensity ratio (mAPP/imAPP) was determined (A). The band intensity of PS1, CTFα and CTFβ was determined, corrected with that of actin (PS1) or holo-APP (CTFα and CTFβ) (A, B) (*n*=3 for APP23-Control and *n*=3 for APP23-GGA). Membrane fractions were subjected to a β- or γ-secretase-mediated peptide cleavage assay as described in the experimental procedures (C) (*n*=8 for APP23-Control and *n*=7 for APP23-GGA). Values are given as mean ± SEM. n.s., not significant.

In general, the production of Aβ is regulated either by the modification of APP or by the modulation of secretase activity. To this extent, we recently reported that the genetic overexpression of HSP70 in mice affects neither the modification of APP nor secretase activity [27]. Here we examined the effects of orally administered GGA on the modification of APP and secretase activity in APP23 mice. The mature (*N*- and *O*-glycosylated) and immature (*N*-glycosylated alone) forms of APP (mAPP and imAPP, respectively) can be differentiated by using SDS-PAGE. As shown in Figure 3A, the total amount of APP and the ratio of mAPP to imAPP in whole cell extracts prepared from the brains of 12-month-old wild-type and APP23 mice were not affected by the oral administration of GGA (Student’s *t*-test, *P* =0.48). We also found that the same treatment did not affect the level of PS1 (Figure 3A) (*n*=3 for APP23-Control and *n*=3 for APP23-GGA) (Student’s *t*-test, *P* =0.34).

The secreted forms of APP generated by α and β-secretase are CTFα and CTFβ, respectively, which can be used as an indirect index of the secretase activity. As shown in Figure 3B, the relative amounts of CTFα and CTFβ were not affected by the administration of GGA to animals (Student’s *t*-test, CTFα, *P* =0.12; CTFβ, *P* =0.42). Under our experimental conditions, the CTFγ band could not be detected (data not shown). We also measured β- and γ-secretase activity directly, using the APP-derived fluorescent substrate. As shown in Figure 3C, the activities of these enzymes were indistinguishable between APP23 mice fed GGA-supplemented chow and those fed control chow (*n*=8 for APP23-Control and *n*=7 for APP23-GGA) (Student’s *t*-test, β-secretase, *P* =0.48; γ-secretase, *P* =0.14). The results in Figure 3 thus suggest that the oral administration of GGA does not affect Aβ production.

**Figure 4:**
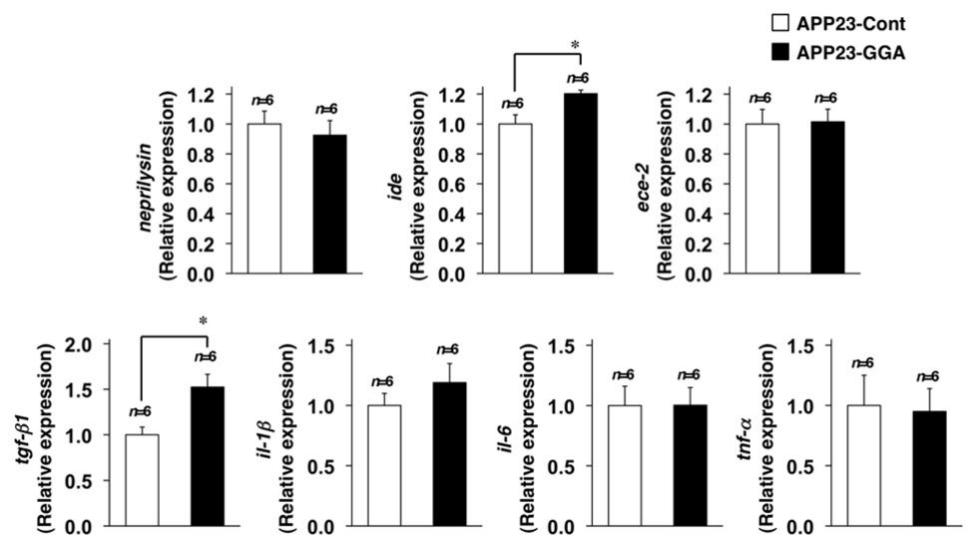
Effects of orally administered GGA on the expression of Aβ-degrading enzymes and cytokines. Total RNA was extracted from the brains of 12-month-old APP23 mice fed either GGA-supplemented chow or control chow (see the legend of Figure 1). Samples were subjected to real-time RT-PCR using a specific primer for each gene. Values were normalized to *gapdh* gene expression and expressed relative to the control sample. Values are given as mean ± SEM (*n*=6 for APP23-Control and *n*=6 for APP23-GGA). *P<0.05.

As described in the Introduction, Aβ degradation by enzymes and Aβ phagocytosis by microglia and astrocytes are involved in the clearance of Aβ [5]. We recently reported that the genetic overexpression of HSP70 affects the expression of genes involved in these processes (*ide* and *tgf-β1*), leading us to examine here the effects of orally administered GGA on the mRNA expression of these genes. Real-time RT-PCR analysis revealed that orally administered GGA up-regulated the expression of *ide* and *tgf-β1* but did not affect the expression levels of the other genes tested (*neprilysin*, *ece-2, il-1β, il-6* and *tnf-α*) (Figure 4) (*n*=6 for APP23-Control and *n*=6 for APP23-GGA) (Student’s *t*-test, *ide*, *P*=0.013; *tgf-β1*, *P*=0.011; *neprilysin*, *P*=0.29; *ece-2*, *P*=0.46; *il-1β*, *P*=0.17; *il-6*, *P*=0.49; *tnf-α*, *P*=0.44). These results suggest that, as for the genetic overexpression of HSP70, orally administered GGA also decreases Aβ levels, at least partly, through the up-regulation of *ide* and *tgf-β1* expression.

### Effects of orally administered GGA on HSP70 expression in the brain

**Figure 5:**
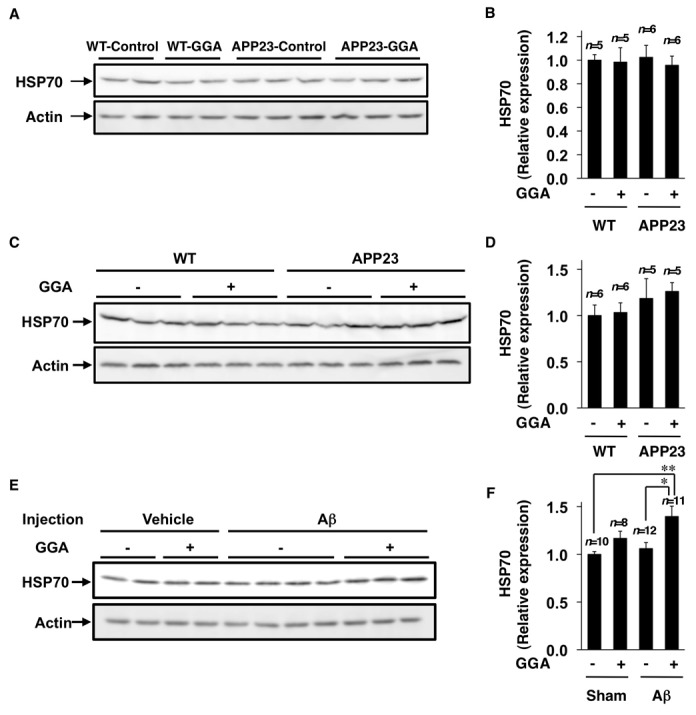
Effects of orally administered GGA and/or injection of Aβ42 oligomers on the expression of HSP70. Wild-type (WT) and APP23 mice were fed either GGA-supplemented chow (10 g GGA/kg chow) or control chow from the age of 3 months to 12 months (A, B) (*n*=5 for WT-Control, *n*=5 for WT-GGA, *n*=6 for APP23-Control, and *n*=6 for APP23-GGA) or from the age of 3 months to 4 months (C, D) (*n*=6 for WT-Control, *n*=6 for WT-GGA, *n*=5 for APP23-Control, and *n*=5 for APP23-GGA). Whole cell extracts were prepared from the brains of 12-month-old mice (A, B) or 4-month-old mice, respectively (C, D). Three-month-old wild-type mice were given a once only orally administered dose of 500 mg/kg of GGA, and 3 h later Aβ42 oligomers (1 µl, 100 pmol) was injected into the hippocampus. Whole cell extracts were prepared from the hippocampus 24 h after the injection (E, F) (*n*=10 for Sham-Vehicle, *n*=8 for Sham-GGA, *n*=12 for Aβ-Vehicle, and *n*=11 for Aβ-GGA). Samples were subjected to immunoblotting with an antibody to HSP70 or actin (A, C, E). The band intensity of HSP70 was determined, corrected to that of actin and expressed relative to the control (B, D, F). Values are given as mean ± SEM. **P<0.01; *P<0.05.

Finally, we examined the effect of orally administered GGA on the expression of HSP70 in the brain under conditions in which alterations of functional and pathological phenotypes in APP23 mice were observed (GGA administration from the age of 3 to 12 months). As shown in Figure 5A and B, this regime of GGA administration did not affect the expression of HSP70 in either APP23 or wild-type mice at the age of 12 months (*n*=5 for WT-Control, *n*=5 for WT-GGA, *n*=6 for APP23-Control, and *n*=6 for APP23-GGA) (Figure 5B) (two-way ANOVA, genotype effect *F*_(1, 18)_=0.01, *P*=0.912; GGA effect *F*_(1, 18)_=0.23, *P*=0.635; interaction *F*_(1, 18)_=0.07, *P*=0.794). We therefore examined the effect of oral GGA administration on the expression of HSP70 under other conditions. First, we considered the possibility that GGA might up-regulate the expression of HSP70 in younger mice (i.e. 3 months old) but not in older ones (i.e. 12 months old) and examined the expression of HSP70 in 4-month-old mice after a 1-month period of oral administration of GGA. As shown in Figure 5C and D, although there was a tendency for GGA to increase the expression of HSP70 in the brain under these conditions, the result was not statistically significant (*n*=6 for WT-Control, *n*=6 for WT-GGA, *n*=5 for APP23-Control, and *n*=5 for APP23-GGA) (Figure 5D) (two-way ANOVA, genotype effect *F*_(1, 18)_=6.11, *P*=0.024; GGA effect *F*_(1, 18)_=0.29, *P*=0.598; interaction *F*_(1, 18)_=0.04, *P*=0.839). We then considered that GGA might require more stressful conditions to up-regulate the expression of HSP70. On this basis, we examined the effect of orally administered GGA and/or the bilateral injection of Aβ42 oligomers into the hippocampus on the expression of HSP70 in this region. As shown in Figure 5E and F, the GGA significantly increased the expression of HSP70 in the brain when Aβ42 oligomers was concomitantly injected into the hippocampal region. The oral administration of GGA, or the injection of Aβ42 oligomers alone, both showed a tendency to increase the expression of HSP70; however, the increase was not statistically significant (Figure 5E and F) (*n*=10 for Sham-Vehicle, *n*=8 for Sham-GGA, *n*=12 for Aβ-Vehicle, and *n*=11 for Aβ-GGA) (Figure 5F) (two-way ANOVA, Aβ injection effect *F*_(1, 37)_=10.32, *P*=0.003; GGA effect *F*_(1, 37)_=45.15, *P*<0.001; interaction *F*_(1, 37)_= 4.11, *P*=0.048). These results suggest that orally administered GGA could up-regulate the expression of HSP70 in the brain in the presence of elevated levels of Aβ oligomers.

## Discussion

As outlined in the Introduction, numerous beneficial effects of HSP70 in animal models of various diseases have been reported. With respect to a range of neurodegenerative diseases, the overexpression of HSP70 has been shown to suppress the aggregation of pathogenic proteins and to ameliorate the corresponding disease symptoms [22,27,36,38]. Such findings give rise to the notion of the potential benefits of HSP70 inducers to retard or inhibit the progression of neurodegenerative diseases. It was reported that orally administered GGA ameliorates disease symptoms in an animal model of spinal and bulbar muscular atrophy [36] and we here examined the effect of GGA on the phenotypes exhibited by an animal model of AD.

It was previously reported that GGA achieves its anti-ulcer activity after absorption by the intestinal mucosa and also that GGA is able to pass through blood brain barrier [39,40]. These findings suggest that GGA could be administered orally to up-regulate the expression of HSPs in the brain. To this extent, in the animal model of spinal and bulbar muscular atrophy mentioned above, the up-take of GGA incorporated in chow (1%) induced the expression of HSP70 in the spinal cord and ameliorated the neuromuscular symptoms displayed by these animals [36]. Moreover, in an animal model of focal cerebral ischemia, orally administered GGA (1000 mg/kg) induced the expression of HSP70 in the brain and reduced the infarct volume [41], while in an experimental intracerebral hemorrhage model, orally administered GGA (800 mg/kg) induced the expression of HSP70 and reduced the extent of hemorrhage [42]. It should be noted that in all these previous reports, the GGA-induced expression of HSP70 was observed in the disease models but not so clearly in control animals. It could thus be interpreted that GGA may enhance the expression of HSP70 induced by other stressors in the brain.

We showed that orally administered GGA improved not only cognitive function (spatial learning and memory) but also reduced the pathological phenotypes (reducing levels of Aβ (monomer and oligomers), Aβ plaque deposition and synaptic loss) exhibited by APP23 mice. We also found that the orally administered GGA did not alter APP maturation or secretase activity in the brain; however the expression of *ide* and *tgf-β1* was up-regulated by this treatment protocol. All these phenomena are similar to those observed in APP23 mice crossed with transgenic mice overexpressing HSP70 [27], suggesting that GGA achieves these ameliorative effects through the up-regulation of HSP70 expression in the brain. In other words, we consider that HSP70’s abilities to unfold and refold Aβ aggregates, to protect against Aβ neurotoxicity, to suppress inflammation and to stimulate Aβ clearance are involved in these ameliorative effects of GGA. However, it should be noted that the extent of amelioration was not so apparent in APP23 mice fed GGA-supplemented chow as in those crossed with transgenic mice expressing HSP70 [27], possibly reflecting the higher level of HSP70 expression in the latter mice.

Surprisingly, orally administered GGA (from the age of 3 to 12 months) did not affect the expression of HSP70 at the brain level even in APP23 mice at the age of 12 months. It was reported that in an animal model of Huntington’s disease, the induction of HSP70 became less apparent as the disease progressed [43]. Thus, considering the possibility that GGA could up-regulate HSP70 expression in younger mice, we examined HSP70 expression in 4-month-old animals after a 1-month period of oral administration of GGA. Although we identified a tendency for this treatment to increase HSP70 expression in the brain, the level of alteration was not statistically significant. We then considered that GGA might up-regulate the expression of HSP70 only in the region confined to where toxic Aβ levels are high, or that Aβ’s neurotoxicity might be so weak that the GGA-dependent up-regulation of HSP70 expression might be undetectable by the immunoblot assay technique. In any case, we predicted that a large increase in Aβ neurotoxicity would cause the GGA-dependent up-regulation of HSP70 expression to become clearly apparent. Indeed, in the presence of Aβ42 oligomers injected directly into the hippocampus, concomitantly administered GGA significantly increased the expression of HSP70.

We cannot be certain at this point in time whether GGA achieves its ameliorative effects on AD-related phenotypes in APP23 mice through its HSP-inducing activity. To this extent, it is possible that GGA achieves its ameliorative effects through HSP-independent mechanisms. Various pharmacological activities of GGA other than HSP-inducing activity have been reported [44,45]. GGA was originally developed as an anti-ulcer drug based on its capacity to increase gastric mucosal blood flow and mucus production [46,47]. We previously reported that GGA protects membranes against NSAID-induced permeabilization [48], and it was recently reported that GGA inhibits replication of the hepatitis C virus through either an inhibitory effect on the geranylgeranylation of proteins or on the activation of mTOR [49,50]. Thus, we cannot rule out the possibility that such HSP-independent pharmacological activities might be involved in the ameliorative effects of GGA on AD-related phenotypes in APP23 mice. Even so, it would be difficult to reconcile such pharmacological activities with the noted improvements in cognitive function, Aβ plaque deposition, synaptic loss, Aβ clearance, Aβ phagocytosis and so on.

Although the molecular mechanism of GGA’s action is not clear at present, the results of this study suggest that GGA could be a candidate for use as a new type of anti-AD drug. A number of new compounds are being developed for this purpose; however, as the development of new molecules requires an enormous economic commitment by drug companies and the distinct possibility exists of encountering unacceptable side effects in late-stage clinical trials. GGA might offer a distinct advantage given that its safety has already been clinically demonstrated.
